# Nature’s Most Fruitful Threesome: The Relationship between Yeasts, Insects, and Angiosperms

**DOI:** 10.3390/jof8100984

**Published:** 2022-09-20

**Authors:** Eduardo D. Fenner, Thamarys Scapini, Mariana da Costa Diniz, Anderson Giehl, Helen Treichel, Sergio Álvarez-Pérez, Sérgio L. Alves

**Affiliations:** 1 Graduate Program in Environment and Sustainable Technologies, Federal University of Fronteira Sul, Campus Cerro Largo, Cerro Largo 97900-000, RS, Brazil; 2 Laboratory of Yeast Biochemistry, Federal University of Fronteira Sul, Campus Chapecó, Chapecó 89815-899, SC, Brazil; 3 Laboratory of Microbiology and Bioprocesses, Federal University of Fronteira Sul, Campus Erechim, Erechim 99700-970, RS, Brazil; 4 Department of Animal Health, Faculty of Veterinary Medicine, Complutense University of Madrid, 28040 Madrid, Spain

**Keywords:** angiosperms, bioprospection, floral nectar, insects, volatile organic compounds, yeasts

## Abstract

The importance of insects for angiosperm pollination is widely recognized. In fact, approximately 90% of all plant species benefit from animal-mediated pollination. However, only recently, a third part player in this story has been properly acknowledged. Microorganisms inhabiting floral nectar, among which yeasts have a prominent role, can ferment glucose, fructose, sucrose, and/or other carbon sources in this habitat. As a result of their metabolism, nectar yeasts produce diverse volatile organic compounds (VOCs) and other valuable metabolites. Notably, some VOCs of yeast origin can influence insects’ foraging behavior, e.g., by attracting them to flowers (although repelling effects have also been reported). Moreover, when insects feed on nectar, they also ingest yeast cells, which provide them with nutrients and protect them from pathogenic microorganisms. In return, insects serve yeasts as transportation and a safer habitat during winter when floral nectar is absent. From the plant’s point of view, the result is flowers being pollinated. From humanity’s perspective, this ecological relationship may also be highly profitable. Therefore, prospecting nectar-inhabiting yeasts for VOC production is of major biotechnological interest. Substances such as acetaldehyde, ethyl acetate, ethyl butyrate, and isobutanol have been reported in yeast volatomes, and they account for a global market of approximately USD 15 billion. In this scenario, the present review addresses the ecological, environmental, and biotechnological outlooks of this three-party mutualism, aiming to encourage researchers worldwide to dig into this field.

## 1. Introduction

Pollinating insects forage flowers in order to nourish themselves, and through these forages, plants can be rewarded with pollination. Although this plant–animal relationship has been documented for more than a century [1], only in the last decades has it been demonstrated that these invertebrates are mostly attracted to the chemical signals released by the microorganisms inhabiting the whole flower—especially by yeasts dwelling in the floral nectaries [2,3,4].

About 550 million years ago, in the Paleozoic era, coexistence with animals and plants directly impacted fungi’s chemical and ecological processes so that new relationships were established between these three kingdoms as they co-evolved [5,6,7]. The hemiascomycetous yeasts of the Saccharomycetes class separated from filamentous fungi around 300 to 400 million years ago and then began to adapt to habitats rich in organic carbon. The same goes for primitive wingless hexapods (Collembola, Protura, among others), pterygote insects, and some holometabola, which evolved around the same period as yeasts [5,6,8,9].

The wide diversity of yeasts thus far described as members of the insect microbiota reflects the latter’s diversity, which consequently influences yeast evolution in this habitat [10]. Angiosperms, which appeared at the beginning of the Cretaceous period (125 million years ago), provided high availability of sugar through the nectar produced in their flowers and fruits (especially the fleshy ones) generated as a result of their sexual reproduction. Such sugar abundance in flowers and fruits offered an outstanding habitat for yeast growth, such as *Saccharomyces*, showing the first traces of interactions between yeasts and insects related to flowers and fruits [11,12].

As by-products of their metabolism, yeasts produce volatile organic compounds (VOCs) that can modify insect behavior. When found in substrates that serve as animal food, such as floral nectar, yeasts can access the insect body to be transported on its surface or inside. Thus, the evolution of the ecological characteristics of these microorganisms is intrinsically linked to the interaction between insects and flowering plants [13,14]. On the other hand, it is worth noting that yeast dispersal by insects is older than pollen. Therefore, the ability to attract insects by releasing diverse chemicals was already present in the yeasts associated with ancient angiosperms, which suggests that the insect–yeast relationship also impacted plant evolution [2].

Nectar-dwelling yeasts need to find another place to live in the nonflowering seasons. Indeed, under these circumstances, yeasts may persist in soil from winter to early spring, and, through the winds, they are then dispersed again to plants. However, some of these microorganisms find, in the insect gastrointestinal tract, a more favorable environment to survive until the next flowering season. When flower season arrives, yeasts are again inoculated into the floral nectaries thanks to the foraging activity of pollinators [15,16].

Some species of the genus *Metschnikowia* (Ascomycota) are the predominant yeasts inhabitants of floral nectar, being found associated with flowers of phylogenetically diverse plants on all continents of the world except Antarctica [17,18,19,20,21,22,23,24,25,26]. Notably, some *Metschnikowia* species show a restricted biogeographic distribution, and their relationships with specific insects and plants may have favored their allopatric or peripatric speciation; that is the case, for example, for *Metschnikowia hawaiiensis*, a yeast species which is exclusively found in association with some plants from Hawaii (USA), and *M. arizonensis*, which has been only found in the USA, Costa Rica, Brazil, and Belize [27,28].

In the last ten years, several new yeast species of the *Metschnikowia* clade have been discovered associated with insects and angiosperms. To name just a few examples, *M. proteae* [29], *M. drakensbergensis*, and *M. caudata* [30] were found to be nectarivorous yeasts associated with *Protea* flowers in South Africa. Still on the African continent, in Morocco, a taxonomic study of the floral nectar yeasts of *Teucrium pseudochamaepitys*, *Teucrium polium*, and *Gladiolus italicus* described the new species *M. maroccana* [23]. In the Cerrado ecosystem (Brazil), Rosa et al. [31] isolated twelve strains of *M. cerradonensis* from flowers of *Ipomoea carnea* and from beetles of the genus *Conotelus*. In the Amazonia, a new species closely related to *M. arizonensis* was discovered in passion fruit (*Passiflora edulis*) flowers and was named *M. amazonensis* [25]. From mustard (*Brassica rapa*) and broad bean (*Vicia faba*) fields in Japan, four new strains closely related to the Hawaiian *M. hawaiiana* were isolated; the four strains were described as a new species named *M. miensis* [24]. Regardless of continent or habitat, there is an extraordinarily high diversity of yeast species that transit between the floral nectaries of diverse plant species and insects. Table 1 lists some such yeast species and the plant and insect taxa with which they are associated.

It is well known that, through their fermentation activity, yeast communities play significant ecological roles in plant reproduction through mutual relationships with pollinator attraction [32,33]. However, some plants pollinated by invertebrates have their nectar sugars primarily metabolized by the dense populations of yeasts in these environments, drastically reducing pollinators’ food reward [34,35]. The following sections will present (i) an overview of the nectar nutrients found in different plant species, (ii) the metabolic pathways carried out by the yeasts inhabiting floral nectar, and (iii) the biotechnological potential of these yeasts. Although it is widely known how beneficial the relationship is to the three parts in question here (yeasts, insects, and plants), we will also address mutualistic relationships in pairs—that is, the mutualism established between yeasts and only one of the other two parties (i.e., plant or insect, but not both simultaneously). Finally, this article aims to encourage researchers worldwide to dig into yeast prospection for high-added-value VOCs.

**Table 1:** Examples of nectar- and insect-associated yeast species.

Yeasts	Plants	Insects	References
*Candida ipomoeae*	*Ipomoea* spp. and Convolvulaceae	*Conotelus* sp.	[22]
*Candida kunwiensis*	*Ipomoea batatas*	*Bombus terrestris*	[36]
*Candida powellii and Candida tilneyi*	*Ipomoea carnea*	*Conotelus* sp.	[26]
*Cryptococcus albidus*	*Helleborus foetidus*	*Bombus* spp.	[15]
*Cryptococcus victoriae*	*Helleborus foetidus*	*Bombus* spp.	[15]
*Cystofilobasidium capitatum*	*Helleborus foetidus*	*Bombus* spp.	[15]
*Kodamaea transpacifica*	*Ipomoea alba*	Beetles (*Nitidulidae)*	[37]
*Metschnikowia amazonensis*	*Passiflora edulis*	*Conotelus* sp.	[25]
*Metschnikowia bowlesiae*	*Ipomoea indica*	*Conotelus mexicanus*	[27]
*Metschnikowia caudata*	*Protea roupelliae*, *Protea dracomontana*, and *Protea subvestida*	*Apis mellifera*	[30]
*Metschnikowia cerradonensis*	*Ipomoeae carnea*	Beetles (*Conotelus*)	[31]
*Metschnikowia cubensis*	*Ipomoea acuminata*	*Conotelus* spp.	[38]
*Metschnikowia drakenbergensis*	*Protea dracomontana*	*Heterochelus* sp.	[30]
*Metschnikowia drosophilae*	*Ipomoea* sp.	*Drosophila bromeliae*	[26]
*Metschnikowia lochheadii*	*Ipomoea indica*	*Conotelus mexicanus*	[26]
*Metschnikowia maroccana*	*Teucrium polio*	NR *	[23]
*Metschnikowia miensis*	*Brassica rapa*	NR *	[24]
*Metschnikowia proteae*	*Protea caffra*	*Atrichelaphinis tigrina*, *Cyrtothyrea marginalis*, and *Heterochelus* sp.	[29]
*Metschnikowia reukaufii*	*Helleborus foetidus*	*Bombus terrestris*	[39]
*Metschnikowia santaceciliae, Candida hawaiiana,* and *Candida kipukae*	*Ipomoea indica*	*Conotelus* spp.	[40]
*Metschnikowia vanudenii*	*Asclepias syriaca*	Flies (*Muscidae*)	[20]
*Pseudohyphozyma bogoriensis*	*Lamprococcus chlorocarpus*	Bees	[41]
*Pseudozyma hubeiensis*	*Cryptanthus dianae*	Bees	[41]
*Sporobolomyces carnicolor*	*Aechmea froesii*	Bees	[41]
*Teunia rosae*	*Rosa chinensis*	NR *	[42]
*Teunia rudbeckiae*	*Rudbeckia bicolor*	NR *	[42]

* NR = not reported by the authors.

## 2. Yeasts at Work: Nectar Fermentation and VOC Production

### 2.1. Nectar Composition

For pollen dispersal to occur between plants, about 90% of angiosperm species rely on animals that forage flowers in search of food resources [43,44,45,46]. From the pollinator’s point of view, preferences for flowers take into account the amount of nectar available, the variability and concentration of its nutrients, and even the microorganisms found in the nectary [47,48,49].

**Figure 1:**
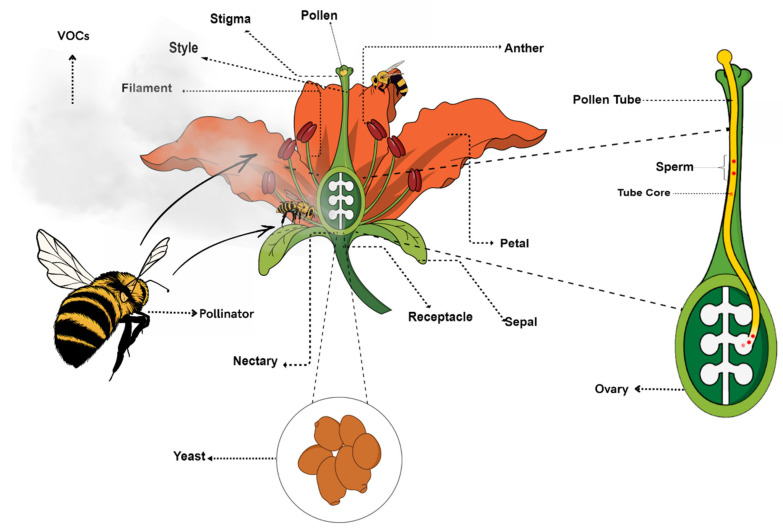
Yeasts in flower nectaries ferment sugars, metabolize amino acids, and produce VOCs that might alter insects’ behavior. When these invertebrates feed on nectar, pollen is transported from anthers to the stigma (which may happen between different individual plants or between both parts in the same flower). Then, pollen germinates, and the pollen tube emanates, eventually allowing fertilization.

Nectar is produced in the nectariferous gland, found in angiosperm flowers either at their receptacles, hypanthia, petals, sepals, stamens, or pistils (Figure 1), and its primary function is to attract animal pollinators, including insects [50,51]. Nectar is a sugar-rich aqueous solution whose composition varies widely according to the time and sexual phase of the plant, the environmental conditions, and the pollinator’s activity. Nectar carbohydrates can be found in the form of disaccharides or monosaccharides. Sucrose is the main disaccharide found, while glucose and fructose are the main hexoses [52,53,54,55]. The presence of these compounds is also quite variable across plant species, among species of the same genus, and even within plants of the same species (see Table 2). In addition to carbohydrates, nectars also have other components, but in smaller amounts, such as amino acids, proteins, minerals, protein, non-protein antioxidants, phenols, alcohols, and alkaloids [55,56,57,58,59,60].

### 2.2. Main Metabolic Routes for Nectar-Based VOC Production

#### 2.2.1. Carbohydrate Metabolism

**Figure 2:**
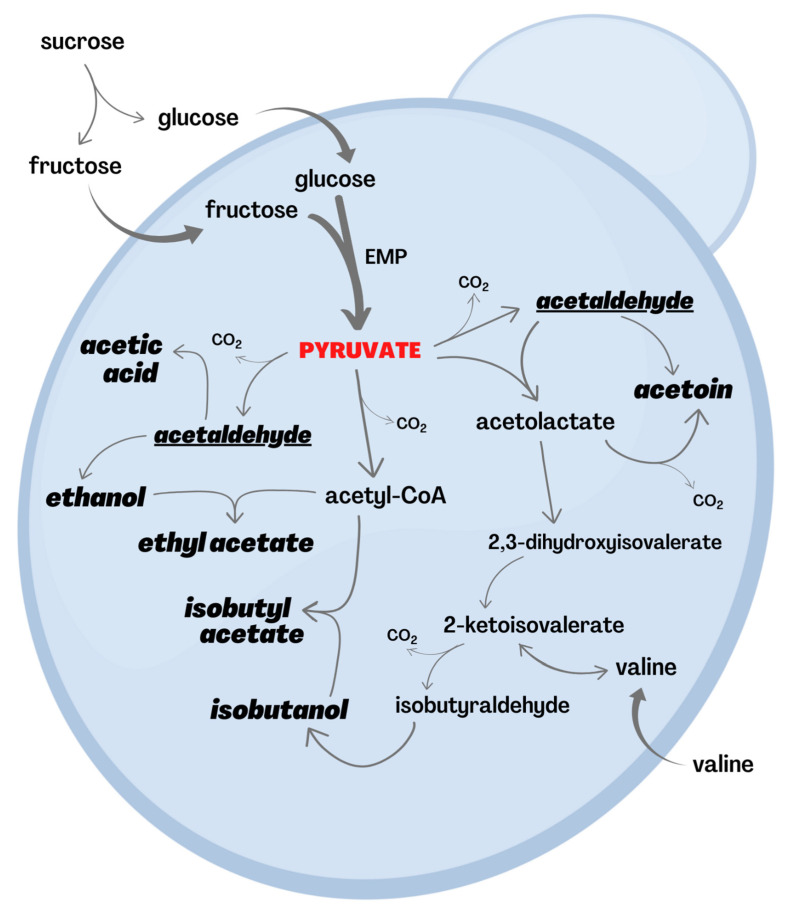
Pyruvate’s central role in VOC production from sugars occurring within the cells of *Saccharomyces cerevisiae* and other model yeasts. The hexoses glucose and fructose can be directly available in nectar or be generated from sucrose hydrolysis. Each mol of these hexoses is converted into two mols of pyruvate through the Embden–Meyerhof–Parnas (EMP) pathway, herein represented by a thick arrow. Pyruvate also works as a precursor of amino acids, such as valine, which can be found in nectar as well. VOCs are shown in bold italics. Acetaldehyde (underlined) can be either secreted as any other volatile compound or converted into different VOCs. Enzymes and coenzymes were omitted for the sake of simplification. Carbohydrate metabolism in *Metschnikowia* and other non-*Saccharomyces* yeasts prevalent in floral nectar remains greatly understudied. For further details, see the main text.

The products of yeast fermentation of sugars are widely described in the literature, although most research on this topic refers to *Saccharomyces cerevisiae* and other model yeasts that are rarely isolated from floral nectar (however, see Gonçalves et al. [69,70,71] for some pioneer studies on the carbohydrate metabolism of the *Wickerhamiella/Starmerella* clade, which is prevalent in floral nectar). In any case, it is known that, depending on the yeast species involved and the environmental conditions, many VOCs may be obtained from carbohydrate metabolism [72,73,74]. This is especially relevant for acetaldehyde, ethanol, ethyl acetate, acetic acid, and acetoin, which can be produced within one-to-three reactions (of a metabolic pathway) from pyruvate [75,76,77,78,79,80]. In fact, as an intermediate product of sugar oxidation, pyruvate can work as a wildcard and be used in different metabolic pathways (Figure 2).

Sucrose, glucose, and fructose are the major sugars in nectar [55,68,81]. Furthermore, yeast cells may hydrolyze sucrose either in the periplasm, cytoplasm, or both [82]. Glucose and fructose result from this breakdown, and then they are channeled to the Embden–Meyerhof–Parnas (EMP) pathway, producing two mols of pyruvate for each mol of monosaccharide. Pyruvate can follow the alcoholic fermentation route, being decarboxylated into acetaldehyde, which, in turn, is predominantly reduced into ethanol. Nevertheless, acetaldehyde may have two major species-dependent alternative fates that matter in this context: acetic acid and acetoin. While the first results from acetaldehyde oxidation [77], the second emerges from the condensation of two acetaldehydes. Moreover, when acetaldehyde eventually condensates with pyruvate, acetolactate arises, and this later compound can also be decarboxylated into acetoin [79]. Besides acetoin, acetolactate may give rise to isobutanol as well. In this case, though, it must be first reduced into 2,3-dihydroxyisovalerate, and this is then dehydrated to 2-ketoisovalerate. In turn, 2-ketoisovalerate can be either aminated, producing valine, or decarboxylated into isobutyraldehyde, which is reduced into isobutanol [75,77,80]. Finally, pyruvate can also be oxidized and decarboxylated into acetyl coenzyme A (acetyl-CoA). When it alternatively condensates with ethanol or isobutanol, acetyl-CoA generates ethyl acetate or isobutyl acetate, respectively [78,80] (Figure 2).

Floral nectar can also contain glycoconjugates formed by monosaccharides bound (especially through a ß-glucosidic linkage) to aromatic compounds (aglycones), such as geraniol [83,84], α-terpineol [85,86], methyl salicylate [85,87], 1-hexanol [88,89], eugenol [59,90], vanillin, and vanillyl alcohol [91,92,93]. When these glycoconjugates are hydrolyzed by yeast glucosidases, their aglycones volatilize [94,95,96,97,98], working as attractive or repelling agents for insects (see Section 3).

Last but not least, it is worth noting that pyruvate is an α-keto acid, from which different amino acids can result. This is not just the case of valine, as shown in Figure 2, but also of leucine, isoleucine, and alanine, which can be produced when pyruvate reacts with itself, oxaloacetate (another α-keto acid), threonine, or glutamate [99,100]. The following section will address the role of amino acid metabolism in VOC production.

#### 2.2.2. What Else, Besides Sugar, May Nectaries Offer to Yeast-Based VOC Production?

Although VOCs can be mostly produced from sugar metabolism, as stated before, other nutrients available in floral nectar cannot be disregarded in the generation of volatile compounds. In fact, these nutrients are meant to work as precursors, being sometimes converted into VOCs. Considering the diversity of the compounds besides sugars in natural nectars (Table 2), amino acids stand out as the second most abundant components [65,101,102], and their transformation increases the myriad of VOCs that may be produced in nectaries [3,76]. As an additional evolutionary strategy, a high rate of tandem gene duplication in the genome of the prevalent nectar-dwelling yeast *Metschnikowia reukaufii* has been reported [103]. It is worth noting that those duplicated genes are directly related to the improvement of this yeast nitrogen metabolism. Moreover, Dhami et al. [103] found that the high-capacity amino acid importers encoded by *GAP1* and *PUT4* genes were highly expressed in synthetic nectar and regulated by the availability and quality of amino acids. Interestingly, the rapid depletion of nitrogen sources promoted by gene duplications is, in fact, a key mechanism of the priority effects that determine the co-occurrence of nectar microbes.

When metabolizing amino acids, yeasts produce higher alcohols (alcohols that have more than two carbons) and esters [104,105]. Although they are present in much less concentration than sugars [43,55,68,106], virtually all proteogenic amino acids (those twenty used in the protein translational process) are found in floral nectars [66,101,106], with proline, phenylalanine, histidine, asparagine, serine, glutamine, cysteine, and glutamate being the most prevalent ones [65,68,101,102,107,108]. Among them, phenylalanine and cysteine can be, respectively, converted into the higher alcohols 2-phenylethanol and 2-mercaptoethanol through the three-step (transamination–decarboxylation–reduction) Ehrlich Pathway. Moreover, when a higher alcohol such as 2-phenylethanol reacts with acetyl-CoA (which can be originated through both sugar and amino acid oxidation), an acetate ester such as 2-phenylethyl acetate can be easily produced, contributing to the formation of the floral bouquet (reviewed by Dzialo et al. [76]).

In addition to these VOCs, it is worth mentioning that other amino acids (yet present in lower concentrations in floral nectar) can also be converted, within a few reactions, into several other aroma compounds. This is the case of branched-chain amino acids (BCAAs), such as valine (see Figure 2). When catabolized, BCAAs result in their α-keto-acid derivative branched alcohols 2-methyl propanol (isobutanol) and 2- and 3-methyl butanol (isoamyl alcohol) [109,110]. Moreover, threonine, an amino acid highly metabolized by yeasts [76,111,112], may be converted into propanol, butanol, propionic acid, acetic acid, and ethyl acetate [113].

Among the amino acids typically found in nectar, some are not used to build proteins (i.e., the so-called non-protein amino acids—NPAAs). About 250 NPAAs have already been found in plants, especially in the families Fabaceae, Sapindaceae, and Cucurbitaceae [114]. Although NPAAs play central physiological roles in plants, mainly as bioactive compounds (acting as antiherbivore, antimicrobial, antioxidant, and/or growth-promoting agents), some of these amino acids are known to be metabolized by yeasts, namely, taurine [115], β-alanine [116,117], citrulline [89,118], ornithine [119], γ-aminobutyric acid (GABA) [120,121], hydroxytryptophan [122], selenocysteine [123], methionine sulfoxide [124], serotonin [125], and dopamine [126]. Despite the lack of studies regarding VOC production from NPAAs by nectar microbes, it is likely that some of them (especially those with S-containing side chains) are converted into volatile compounds by yeast cells [127].

## 3. Double Agent Yeasts

The presence of yeasts in flowers and insects has been acknowledged since the 19th century (see, for example, the pioneer study of Boutroux [128]), but it is during the last few decades that the mycological study of flowers and their pollinators has acquired a particular interest [15,21,22,129,130,131]. In most cases, the relationship between plants and nectar yeast has been pointed out as beneficial to angiosperms because these microorganisms contribute to attracting pollinating insects (as described above). Despite this fruitful symbiosis for the three parts involved, flowers and yeasts can also work together as a duo, establishing a partnership against some insects (e.g., by attracting the parasitoids that infect some pest insects [112,132]).

Nectar-inhabiting yeasts can thus work as “double agents”, either attracting or repelling insects [133]. Although repelling insects may appear to be a disservice to the plant (avoiding approximation of potential pollinators), it may indeed protect the plant from mere pollinivorous insects (the ones that feed on pollen). In line with that, Ljunggren et al. [132] showed that the presence of *Metschnikowia andauensis* and *Metschnikowia pulcherrima*, which are often found in association with insects feeding on foliage, flowers, and/or fruits, had a repelling effect on larvae of the cotton leafworm *Spodoptera littoralis* (Lepidoptera, Noctuidae), an insect species that naturally feeds on the foliage of a wide spectrum of broad-leaved plants [134]. Furthermore, the authors highlighted some VOCs produced by those yeasts in a particularly high manner—such as ethyl 3-methyl butanoate, ethyl propanoate, heptan-4-ol, nonan-2-ol, and sulcatone—which may be involved with the repelling effect they observed.

Interestingly, yeasts may also act against some pest insects, namely, aphids, by attracting their natural parasitoids to the flowers. In this context, Sobhy et al. [112] showed that the aphid parasitoid wasp *Aphidius ervi* is highly attracted by VOCs produced by the nectar specialist yeasts *M. gruessii* and *M. reukaufii*. Moreover, this *Metschnikowia*-fermented nectar proved to offer satisfactory amounts of macro- and micronutrients to meet the *A. ervi* needs. The results found by those authors suggest that parasitoid-attracting VOCs may integrate strategies of insect pest biocontrol.

However, yeasts can also work the other way around, i.e., pro-insect and against plants. In this case, one can say that the plant is the betrayed party. As Ljunggren et al. [132] also showed, the plant-associated yeasts *Cryptococcus nemorosus*, *Metschnikowia lopburiensis*, and *M. hawaiiensis* had an attracting effect on the cotton leafworm *S. littoralis* larvae. It is likely that geranyl acetone, cyclohexanone, 2-ethyl-1-benzofuran, and 1,3,5-undecatriene produced by the so-referred yeasts are related to the leafworm attraction [132]. In this yeast–insect partnership, while the plant is attacked by herbivores, the animal finds a rich nutrient source, and the microorganism benefits from dispersal and a safe breeding place [135,136].

Curiously, Herrera et al. [39] showed that free-ranging bumble bees (*Bombus terrestris*) preferred to feed on yeast-containing nectar of the early-blooming herb *Helleborus foetidus*. However, yeast presence led to a reduction in the number of pollen tubes in style and, consequently, a decline in plant fecundity. In agreement with these findings, *M. reukaufii* has been considered a nectar contaminant. By consuming the nectar sugars, this yeast ends up inhibiting pollen germination (which relies on these carbohydrates as carbon and energy sources) and probably limits fertilization and fruit set in *Asclepias syriaca* [137,138]. The negative effect of *M. reukaufii* on plant fecundity was also corroborated by de Vega and Herrera [139], who demonstrated that the growth of this yeast species renders floral nectar nutritionally poor; the nutrient concentration on flower decreases while yeast density increases. In contrast, Zhou et al. [140] recently showed a positive effect of *Pichia fermentans* on *Carya illinoinensis* pollen germination ability, with an average increase of 33.6%. Additionally, Colda et al. [141] observed significantly higher visitation rates of honeybees and hoverflies to the flowers of different varieties of European pear trees (Pyrus communis) when *M. reukaufii* was inoculated on nectar with the bacterium *Acinetobacter nectaris*. However, this visitation increase was not seen when both microorganisms were separately sprayed in the pear flowers [141].

Finally, de Vega et al. [142] have recently reported that the effects of *M. reukaufii* on the reproduction of different Mediterranean plants ranged from negative to neutral or positive, depending on the plant species. Moreover, the authors suggested that the inter-species variation in the indirect effects of nectar-inhabiting yeasts on plant pollination might be due to the variation in the pollinator community, the specific microbes colonizing floral nectar, and the order of microbial arrival to the nectary (i.e., priority effects) [142]. Therefore, the differences between treatments observed by these authors might be driven not only by the interaction of *M. reukaufii* with insect pollinators, but also by the interactions of this yeast species with other nectar microbes, such as bacteria [142].

Be that as it may, the nutritional decay of floral nectar results in decreased concentrations of sucrose, glucose, and fructose, as well as intraspecific characteristics of the nectar, directly impacting the behavior of pollinators [34,53]. Herrera et al. [129] emphasize that the density of yeast cells in nectar can reach an order of between 10^3^ and 10^5^ cells/mm^3^, which explains such a change. On the other hand, although this consumption of nutrients by nectar yeasts may seem harmful (from the plant’s point of view), it is worth noting that the biotransformations carried out by these microorganisms and even their own cells may be attractive to pollinators and other flower-visiting insects. Furthermore, these metabolic activities produce heat [143,144] and can increase flower temperature by up to 6 °C [145]. This increase in temperature, especially in winter-blooming plants or angiosperms from arctic and alpine environments, has been shown to be beneficial for pollination. In this context, some hypotheses can be raised to explain this benefit: (i) the heat released warms the internal air of the flower and, to a certain extent, the air around it as well, making the temperature more attractive to pollinators; (ii) the increase in temperature favors plant metabolism and, consequently, pollen germination [145]; and (iii) the heat gain facilitates the volatilization of compounds that attract pollinating insects [146].

## 4. Prospecting Yeasts for Biotechnological Purposes

The ecological functions of nectar-inhabiting yeasts and the relevance of their relationship with angiosperms and insects for the environmental equilibrium are unquestionable. Furthermore, from a biotechnological point of view, the exploitation of yeast VOCs emerges as a strategy for process development, since there is an increasing commercial interest and market expansion of these compounds. Acetaldehyde, dimethyl disulfide, ethyl acetate, 2-phenyl ethanol, 3-methyl-1-butanol, 2-acetyl furan, indole, geranyl acetone, hexanoic acid, and benzyl alcohol are some examples of VOCs emitted by nectar-inhabiting yeasts that could be commercially exploited as biotechnological products in fermentative processes [133].

VOCs’ market expansion attracts advances in yeast prospecting research and bioprocess investment for product development. For example, from the aldehyde class, acetaldehyde is highlighted as a VOC widely expanding in the market and which may be of interest for production by yeasts prospected from angiosperms. This compound is considered a building block for its wide industrial application, being used in the manufacture of acetic acid, flavorings, dyes, and medicines, and it is an intermediate compound for the production of different alcohols [147]. Between 2015 and 2021, the market value of acetaldehyde increased from USD 1.14 billion to USD 1.53 billion, and is expected to further increase to USD 2.54 billion by 2029 [148]. Notably, acetaldehyde is commonly reported as a VOC produced by yeasts obtained from floral nectar, such as *M. reukaufii* and *M. gruessii* [88,112]. *Aureobasidium pullulans* also has the potential for acetaldehyde production. This species has also been reported to produce other compounds of interest, such as n-propanol, isobutanol, 2-methyl-1-butanol, and ethanol [88]. Another important characteristic of *A. pullulans* is its ability to assimilate glucose and xylose, which makes it a promising candidate for the co-production of second-generation bioethanol (using hydrolysates from lignocellulosic biomass) [149].

Given their commercial relevance, the alcohols produced by nectar yeasts can expand the field of biofuels and fine chemical production. For example, ethanol is a primary metabolite of yeast fermentative processes, being a widely reported VOC among prospected yeasts of angiosperms, and has a high expanding economic interest [88,112,149]. The compound annual growth rate (CAGR) of ethanol is estimated to increase by 4.8% from 2020 to 2027, with a market value of USD 89.1 billion in 2019 [150]. The application of ethanol as a biofuel is the most widespread. Furthermore, ethanol is considered a building block in the industry, since it can be used in the automotive industry and in the medical and food fields. Besides the potential of *A. pullulans* for xylanase production and ethanol co-production, yeasts prospected from angiosperms, such as *M. reukaufii*, *M. koreensis*, and *Rhodotorula* sp. are also associated with ethanol production [149,151].

Other alcohols of interest detected in these natural systems are, for example, 2-methyl propanol, 2-phenyl ethanol, 3-methyl-butanol, 2-methyl-butanol, and 2-ethyl-1-hexanol [88,112,133,151]. The prospection of VOC-producing yeasts in angiosperms is an alternative for the biotechnological development of a mixture of alcohols with high added value for the biotechnological industry, since these alcohols are used in different sectors for the development of solvents, sanitizing agents, plasticizing agents, and in other highly valued chemicals. The commercial interest of these compounds is associated with the growth of other sectors where these alcohols are building blocks for interesting products, e.g., a 5% growth in the CAGR of 2-ethyl-1-hexanol is estimated between 2020 and 2025. As this alcohol is used in products such as paints, coatings, other construction materials, and adhesives, its market value is projected to expand while its demand increases [152].

A dual role of yeasts in this insect–angiosperm relationship can also be considered. On the one hand, the mutualistic relationship strongly impacts the plant’s nectar chemistry, since yeasts produce VOC mixtures that can attract more insects to the plant. Therefore, the main market interest is focused on prospecting yeasts as VOC production biofactories. On the other hand, this distinctive mixture of VOCs can have a neutral or repelling effect on insects, and exert antimicrobial functions, which can be of interest to exploring the potential of these yeasts and the compounds they produce in agricultural biocontrol [112,153].

The mechanisms of communication between insects and their microbiota have yet to be explored, but there is growing interest in exploiting the insect–microbe system for agricultural biocontrol. These processes may boost studies on the possibilities of using the attraction based on the insect–microorganism system. Since it has been shown that VOCs produced by yeasts can attract hosts to locate food, Sobhy et al. [112] have suggested that strategies to attract the insect out of the planting area would prevent agricultural pest populations from reaching levels of economic damage.

Other strategies such as the production of extracts containing VOC-producing yeast cells or other antimicrobial compounds may also be exploitable. *Metschnikowia pulcherrima* was used for postharvest biocontrol of blue mold infections of apples (caused by *Penicillium expansum*) and was observed to significantly reduce the disease on the fruit during one month of storage, and, to exhibit resistance to diphenylamine, a postharvest antioxidant treatment [154]. Similarly, *Sporobolomyces roseus* was isolated from grape flowers and used for postharvest biocontrol against *P. expansum*, proving to control pathogen growth and mycotoxin production. The results were associated with competition for nutrients and the production of antifungal VOCs [155]. Due to the advances in research on reducing the use of agrochemicals in agriculture, this field of using yeasts related to pollination systems as biocontrol agents for pests in agriculture is still an unexplored gap in research that has a broad biotechnological potential [154,155,156].

The biotechnological potential of yeasts in the foods and beverages industry (for example in bioflavor production) can be achieved through two approaches: by adjusting the environmental factors of fermentation conduction or via genotype modification of yeast strains. Changing environmental factors can be an important and convenient but sometimes challenging strategy to optimize the production of desired compounds. Therefore, given the recent expansion of the biodiversity of newly isolated yeasts, genetic engineering strategies have been driven to select or develop strains with aromatic properties far beyond what is possible by adjusting environmental parameters [76].

The scenario herein described thus makes clear the importance of prospecting new yeast strains for their potential for VOC production, leading to expanding the biotechnological markets. Table 3 summarizes the industrial application of the main VOCs produced by insect–plant-associated yeasts. Although the real industrial potential nectar-inhabiting yeasts remains to be thoroughly evaluated in terms such as process yield vs. production costs, some yeasts species often found in flowers (including floral nectar) and insects, such as *Aureobasidium pullulans*, *Sporobolomyces* spp., and *Yarrowia lypolytica*, have already demonstrated unique metabolic, genetic, and/or physiological features to play a major role in diverse bioprocesses [157,158,159]. All in all, the near future seems promising, since new technologies for evaluating and collecting these compounds have been investigated and could be significant drivers of further research and investments in the field [157].

## 5. Conclusions

It is generally acknowledged that a better understanding of yeast diversity in natural habitats, their tolerance to environmental stressors, their relationships with other organisms, and their ecological roles may help to improve the current biotechnological uses of these microorganisms and develop novel applications. Furthermore, the application of ecological concepts to the design of yeast-based bioprocesses might contribute to achieving the major goals of a circular bioeconomy, namely, the sustainable, resource-efficient valorization of biomass and other resources in integrated production chains [165].

As discussed in previous sections, nectar-inhabiting yeasts have a huge potential in diverse bioprocesses due to the diverse metabolic capabilities linked to niche adaptations they possess and their tolerance to the multiple stress factors often encountered in their natural habitat, such as high osmotic pressure, limited nitrogen availability, acidic pH, presence of toxins of plant origin, and strong competition with other microbes [56,58,61,103,166,167,168,169]. Production of a huge variety of VOCs is an additional desirable trait of some nectar yeasts that opens the door to new strategies for controlled pollination and pest biocontrol [112,141,170]. Therefore, although it might still take a long time to dethrone *Saccharomyces cerevisiae* as the main (and sometimes the only) yeast used in most current bioprocesses, we expect that further research on nectar-inhabiting yeasts might lead to expanding the list of non-conventional yeasts of biotechnological interest [171].

## Figures and Tables

**Table 2:** Nectar composition of different angiosperm species.

Plant Species	Sucrose (%) ^a^	Glucose (%) ^a^	Fructose (%) ^a^	Amino Acids Detected ^b^	References
*Aconitum* spp. ^c^	39.9–87.6	0–2.9	9.5–60.1	-	[52]
*Antirhinum australe*	78.2 ^d^	9.4 ^d^	12.5 ^d^	-	[61]
*Aquilegia* spp. ^c^	96–98.4	1.0–2.5	0.6–1.5	-	[61]
*Billbergia distachia*	69.5	14.8	15.3	Ala, Asp, Arg, Asn, GABA, Gln, Glu, Gly, His, Ile, Leu, Lys, Met, Phe, Ser, Thr, Tyr, Val	[62]
*Cotoneaster* spp. ^c^	0–11.2	22.9–75.0	25.0–65.9	-	[63]
*Diplacus (Mimulus) aurantiacus*	66.6 ^d^	13.3 ^d^	20.1 ^d^	Ala, Arg, Asp, CIT, GABA, Glu, His, Leu, Pro, Ser, Thr, Tyr, Val	[64]
*Gladiolus illyricus*	51.3 ^d^	30.7 ^d^	18.0 ^d^	-	[61]
*Gentiana lutea*	<1.5 ^d^	50.0–55.0 ^d^	45.0–50.0 ^d^	Ala, BALA, Arg, Cys, CIT, L-HSE, GABA	[65]
*Iris* spp. ^c^	71.4–94.1	3.6–18.6	2.3–10.0	-	[61]
*Lonicera* spp. ^c^	63.5–64.6	22.3–22.7	12.7–14.2	-	[61]
*Marrubium supinum*	43.6 ^d^	26.7 ^d^	29.7 ^d^	-	[61]
*Neottia ovata*	18.3	44.0	37.8	Ala, BALA, Arg, Cys, AABA, GABA	[66]
*Nicotiana* spp. ^c^	3.8–57.0	2.7–38.5	29.8–63.2	AABA, Ala, Asn, Asp, BALA, GABA, Gln, Glu, Gly, His, Leu, Lys, Ile, ORN, Phe, Pro, Met, Ser, Thr, Trp, Tyr, Val	[67]
*Polemonium caeruleum*	42.1 ^e^	21.0 ^e^	32.8 ^e^	Arg, BABA, Gln, Glu, His, Ile, Leu, Lys, Met, NVA, ORN, Phe, Pro, Ser, Thr, Val	[68]
*Vicia* spp. ^c^	54.2–56.0	23.9–26.2	19.6–20.1	-	[61]

^a^ Percentages of the amount of sugar, as reported in the references consulted. ^b^ Compounds determined to be present in nectar in the references consulted. Some of them did not determine other compounds besides sugar. AABA, α-aminobutyric acid; AAPA, α-aminoadipic acid; Ala, alanine; Arg, Arginine; Asn, asparagine; Asp, aspartate; BABA, β-aminobutyric acid; BALA, ß-alanine; CIT, citrulline; Cys, cysteine; GABA, γ-aminobutyric acid; Gln, glutamine; Glu, glutamate; Gly, glycine; His, histidine; Ile, isoleucine; Leu, leucine; LHSE, L-homoserine; Lys, lysine; Phe, phenylalanine; Met, methionine; NVA, norvaline; ORN, ornithine; Pro, proline; Ser, serine; Thr, threonine; Trp, tryptophan; Tyr, Tyrosine; Val, valine. Proteogenic amino acids have only the first letter capitalized; non-protein amino acids have all letters capitalized. ^c^ Values are the range of different species of the same genus in each indicated study. ^d^ Approximated mean values based on the different experimental conditions tested by the authors. ^e^ Values are the mean of 14 studied populations by [68].

**Table 3:** Summary of the biotechnological potential of insect–plant-associated yeasts for VOC production.

VOCs	Producing Yeasts	Industrial Application	References
Acetaldehyde	*Aureobasidium pullulans* *Metschnikowia reukaufii* *Sporobolomyces roseus* *Hanseniaspora uvarum* *Yarrowia lipolytica*	Adhesive Corrosion inhibitor Flavoring agent Personal care Pesticide Solvent	[88,112,158,159]
Dimethyl disulfide	*Metschnikowia reukaufii* *Metschnikowia gruessii* *Hanseniaspora uvarum* *Sporobolomyces roseus* *Yarrowia lipolytica*	Flavoring agent	[112,158,160]
Ethyl acetate	*Metschnikowia* *reukaufii* *Sporobolomyces roseus* *Hanseniaspora uvarum* *Aureobasidium pullulans*	Adhesive Household care Flavoring agent Furniture Medical supplies Motor oil Personal and pet care Paint composition Pesticide Pure chemical Solvent	[88,112,158]
Ethanol	*Rhodotorula* sp. *Metschnikowia koreensis* *Metschnikowia reukaufii* *Aureobasidium pullulans* *Yarrowia lipolytica*	Antifoaming agent Antimicrobial active Astringent Defoamer Drying agent Flavoring agent Hand sanitizer Laboratory supplies Personal and pet care Sealant Stabilizing agent Surfactant Solvent	[88,112,133,149,151,158,159,161]
2-phenyl ethanol	*Aureobasidium pullulans* *Hanseniaspora uvarum* *Lachancea thermotolerans* *Metschnikowia reukaufii* *Yarrowia lipolytica*	Flavoring agent Household care Personal care Pesticide Preservative	[88,112,158,162,163]
2-methyl-1-butanol	*Aureobasidium pullulans* *Hanseniaspora uvarum* *Metschnikowia gruessii* *Metschnikowia reukaufii* *Sporobolomyces roseus*	Flavoring agent	[88,112,151,158,161]
2-ethyl-1-hexanol	*Aureobasidium pullulans* *Metschnikowia reukaufii*	Additive Building materials Dispersant Flavoring agent Solvent	[88,151,161]
Indole	*Lachancea thermotolerans* *Yarrowia lipolytica*	Flavoring agent Personal care	[162,164]
Geranyl acetone	*Lachancea thermotolerans*	Flavoring agent	[162]
Hexanoic acid	*Lachancea thermotolerans* *Yarrowia lipolytica*	Household care Cleansing Emulsifying Chemical Personal care Surfactant Solvent	[159,162]
Benzyl alcohol	*Lachancea thermotolerans*	Antimicrobial Adhesive removers Binder Craft supplies Chemical synthesis Cleaning agent Curing agent Emulsifier Flavoring agent Personal and pet care Solvent Surfactant Viscosity modifier	[162]
Acetic acid	*Metschnikowia reukaufii* *Metschnikowia koreensis* *Yarrowia lipolytica*	Antimicrobial agent Craft supplies Buffering agent Flavoring agent Household care Laboratory supplies Pesticide Refining agents	[151,159]
